# Evaluation of narrow band imaging in the assessment of laryngeal granuloma

**DOI:** 10.1038/s41598-019-50699-8

**Published:** 2019-11-06

**Authors:** H. Klimza, W. Pietruszewska, J. Jackowska, K. Piersiala, M. Wierzbicka

**Affiliations:** 1 Department of Otolaryngology, Head and Neck Surgery, Poznań University of Medical Sciences, Poznań, Poland; 2 Clinical Department of Otolaryngology and Oncological Laryngology, Medical University of Lodz, Lodz, Poland; 3 Institute of Human Genetics, Polish Academy of Sciences, Strzeszynska 32, 60-479 Poznan, Poland; 4 Student Research Group at the Department of Otolaryngology, Head and Neck Surgery Poznań University of Medical Sciences, Poznań, Poland; 5 Division of ENT Diseases, Department of Clinical Sciences, Intervention and Technology, Karolinska Institutet, Stockholm, Sweden

**Keywords:** Oral cancer, Predictive markers

## Abstract

Laryngeal granulomas belong to common complications following trans-oral laser microsurgery (TLM). The aim of this study was to evaluate NBI in the differentiation between granuloma-like lesions and local tumor recurrence. 154 consecutive patients after TLM due to early laryngeal cancer were enrolled. In the group, a monthly follow-up including NBI endoscopy was performed. Moderate and severe dysplasia, carcinoma *in situ* and invasive cancer were defined as positive histology, laryngeal granuloma and other benign laryngeal lesions as negative histology and premalignant lesions as suspicious histology. In 47/154 (31%) cases, granuloma-like lesion (GLL) was found. Patients with GLL were divided into two groups based on the NBI classification. In all patients, the microvascular pattern in NBI was compared with the final histology. In group A, with suspicious, perpendicular vessels, 13/13 (100%) samples were positive. In group B, with normal vascular pattern 3/34 (9%) samples were positive and 31/34 (91%) samples were negative. There was a significant correlation between the positive NBI vascular pattern and the final histology (p = 0.00001). Sensitivity, specificity, accuracy of NBI were as follows: 81%, 100%, 94%, respectively.Based on our results, NBI can reliably differentiate between postoperative laryngeal granuloma and local tumor recurrence. In such a manner, this method is very helpful in the follow-up of tumor patients.

## Introduction

Trans-oral laser microsurgery (TLM) is a well-known and widely used method in the treatment of early and moderately advanced glottic cancer. It is characterized by a high rate of local control, five-year survival and five-year disease-free survival^1,2^. However, TLM is associated with a risk of both major postoperative complications such as bleeding or dyspnoea and minor complications such as granulomas, laryngeal webbing and scaring.

Laryngeal granulomas (LG) belong to late, minor, postoperative complications following TLM. LG and glottic web constitute 22% of all complications and affect more likely patients after TLM involving the anterior commissure (AC)^3^. LG are mucosal lesions consisting of granulation tissue and arise as tissue’s response to injury and irritation^4^. Lee *et al*. proved that the frequency of aforementioned complications following TLM depends on surgeon’s experience and the size of the tumor^2^. Most often, LG occurs within 1 or 2 months after surgery^4^. According to the literature, there is no dedicated treatment for LG. There are two approaches: (1) conservative therapy (antireflux therapy, voice therapy, botulin toxin, mitomicin C, steroids) and (2) surgery^5^. Proton pump inhibitors (PPIs) are widely used to treat LG, as laryngopharyngeal reflux is present in up to 76% of affected patients^5–7^. Surgical methods, CO_2_ laser or cold instruments, are faster than conservative therapy, however, they involve a significant risk of local scarring and recurrence. Surgery is traditionally reserved for persisting cases^5^. Although LG are purely benign lesions, there is always a concern of the cancer recurrence within LG or in the surrounding tissue.

In our study, we want to address a question, whether narrow-band imaging (NBI) is useful in the assessment of patients with granuloma-like lesion following TLM due to glottic cancer. NBI is a reliable blue light endoscopy, which is widely used in the detection of early head and neck cancers by assessing mucosal vascular pattern. Thereby, the aim of this study was to evaluate NBI in the differentiation between postoperative laryngeal granuloma and local tumor recurrence.

## Material and Method

We enrolled 154 consecutive patients with confirmed diagnosis of laryngeal cancer, who underwent TLM cordectomy between April 2015 and November 2017 in two tertiary referral centres in Poland: Poznan 114/154 (74.03%) and Lodz (40/154 (26.97%) Medical University Department of Otolaryngology. Demographic and clinical characteristics of enrolled patients are presented in Table 1.

**Table 1:** Demographic and clinical characteristics of enrolled patients.

Variable	All patients	Patients with LG	P value
	N (%)	N (%)	
Sex			
Male	138 (90)	41 (87)	0.522^a^
Female	16 (10)	6 (13)	
Smoking history			
Current heavy smoker	64 (42)	23 (49)	0.218^a^
Former or never smoker	90 (58)	24 (51)	
T-status			
Tis	22 (15)	3 (7)	0.191^a^
T1a	44 (29)	12 (26)	
T1b	24 (16)	8 (17)	
T2	61 (40)	23 (50)	
Cordectomy type			
I	14 (9)	2 (4)	0.373^a^
II	15 (10)	3 (6)	
III	26 (17)	6 (13)	
IV	22 (14)	9 (19)	
V	43 (28)	14 (30)	
VI	34 (22)	13 (28)	
Treating Department			
Poznan	114 (74)	36 (77)	0.630^a^
Lodz	40 (26)	11 (23)	
Location of LG			
AC		22 (47)	—
PG		20 (43)	
MG		5 (10)	

^a^Chi2 test.

Following TLM, all patients were scoped once a month with trans-nasal flexible video-endoscope (Olympus Medical System Corporation, Tokyo, Japan) by means of white light, NBI and video-stroboscopic evaluation.

In 47/154 patients (31%) granuloma-like lesion (GLL) was found. In 22 cases (47%), it was localised in the anterior commissure, 20/47(43%) in posterior one third of the glottis and 5/47 (11%) in the middle part of the glottis (Table 1).

First, each patient with GLL underwent trans-nasal flexible video-endoscope (Olympus Medical System Corporation, Tokyo, Japan) with the optical filter for NBI. By using blue light (NBI), we assessed the vascular pattern of glottis and in particular vascular pattern of granulation tissue and mucosa surrounding the lesion. The diagnostic criteria defining suspected character of the lesion in NBI endoscopy were as follows: symmetrical arranged brown dot-like loops with narrow-angled turning point, which correspond to perpendicular vascular changes according to European Laryngological Society (ELS) classification^8^.

Detecting perpendicular vascular changes during NBI, in or surrounding granulation tissue, classified those patients to the “suspicious” group A. The remaining patients, presenting with longitudinal vascular pattern, which is considered normal, were qualified to the group B. Following NBI endoscopy, excisional biopsies with a cold instrument under general anaesthesia were performed in all patients. Eventually, the surgical specimens were sent to pathology. Moderate and severe dysplasia, carcinoma *in situ* and invasive cancer were defined as positive histology, laryngeal granuloma and other benign laryngeal lesions as negative histology and premalignant lesions as suspicious histology.

The main predictive variables were mucosa and submucosa vascular pattern in patients with GLL (according to ELS classification) and final histology. The additional variables were age, sex and number of days following TLM to GLL detection.

The primary outcome measure was the correlation between the observed vascular pattern of mucosa (according to ELS classification) surrounding GLL and the result of the final histology.

### Ethical approval

All procedures performed in studies involving human participants were in accordance with the ethical standards of the institutional and national research committee and with the 1964 Helsinki declaration and its later amendments or comparable ethical standards. The study protocol was approved by Bioethics Committee of Poznan University of Medical Sciences.

### Informed consent

Informed written consent was obtained from all individual participants included in the study.

## Result

**Figure 1:**
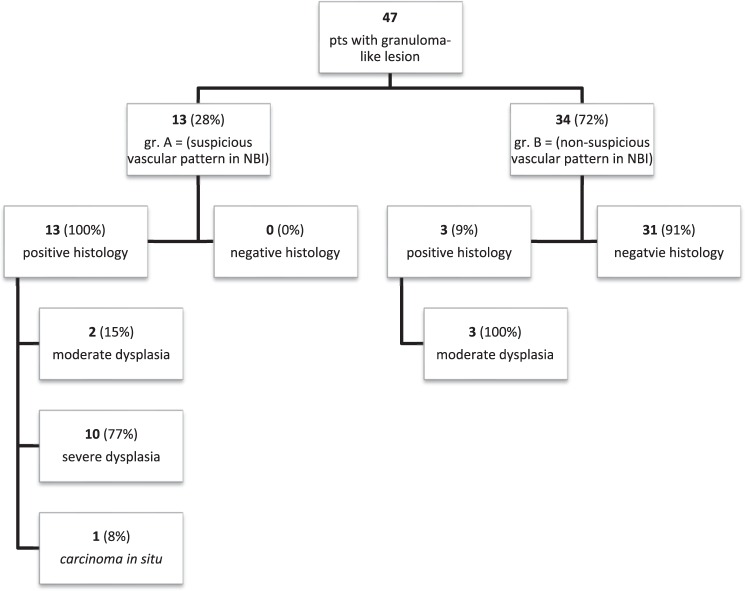
Granuloma-like lesion with the suspicious vascular pattern in NBI.

**Figure 2:**
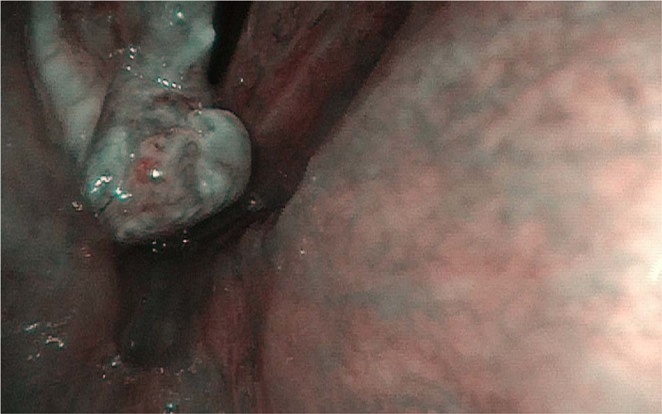
Granuloma-like lesion with the benign vascular pattern in NBI.

Out of 47 patients, who presented with GLL, 13/47 (28%) (group A) had perpendicular vascular changes visualized in NBI on the mucosa surrounding granulation tissue (Fig. 1). The remaining 34/47 (72%) (group B) had longitudinal vascular changes on mucosa surrounding GLL (Fig. 2). In all patients, the microvascular pattern in NBI was compared with the final histology.

**Figure 3:**
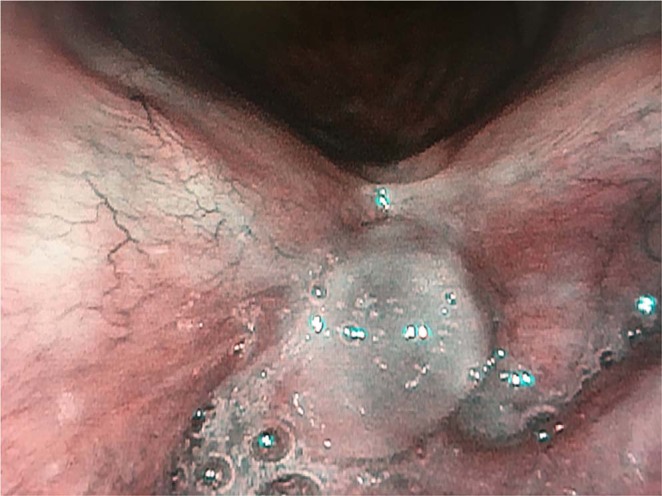
The summary of results.

In group A, with suspicious, perpendicular vessels seen in NBI, the final histology results were: 13/13 (100%) samples were positive: 10/13(77%) severe dysplasia, 1/13(8%) cancer *in situ*, 2/13(15%) moderate dysplasia. (Graph 1.) In group B, with longitudinal vessels seen in NBI, the final histology results were: 3/34 (9%) samples were positive: 3/3: moderate dysplasia, 30/34 samples were negative, all samples were granulation tissue (Fig. 3).

There was a significant correlation between the vascular pattern of the mucosa in NBI in area of the granuloma tissue and the final histological result: Chi2(1) = 34.82; p = 0.00001 (Table 2). Sensitivity, specificity and accuracy of NBI were as follows: 81%, 100%, 94%.

**Table 2:** Chi-square analysis of the NBI vascular pattern vs histology result.

	Positive NBI vascular pattern	Negative NBI vascular pattern
Positive histology	13	3
Negative histology	0	31
	Chi2(1) = 34.82; p = 0.00001	

The local recurrence of laryngeal cancer after excisional biopsy was found in 1/47 (2%) patient during 10^th^ month of follow-up. In presented group, the mean number of days to detection of GLL was 164 days. The earliest detected GLL was visualized after 10 days and the latest after 577 days following TLM. There was no statistically significant correlation between presence of GLL and sex, smoking history, initial T-status, type of cordectomy or treating department (Table 1).

## Discussion

Narrow band imaging has been in use in laryngology for 10 years now. In our previous papers, we proved that the NBI sensitivity, specificity and accuracy in detection of premalignant and malignant head and neck tumors is higher compared with classical endoscopy with white light only^9,10^. At first, NBI positive findings were identified as well-demarcated areas with scattered brownish spots^11,12^. In 2011, Ni *et al*. introduced the NBI classification for premalignant and malignant lesions in larynx. This classification consists of five types of different vascular patterns; additionally type 5 is divided into three subtypes. Types 1–3 are identified as vascular pattern characteristic for benign lesions, whereas types 4–5 are common in premalignant and malignant lesions^13^. However, the aforementioned classification is difficult to implement in everyday practice. Therefore, European Laryngeal Society (ELS) in 2016 proposed simpler classification for glottic lesions^8,14^. Authors distinguished only two types of vascular patterns. The first type is characterized by longitudinal vessels in two dimensions (length and width) and represents benign lesions. The second type, which are perpendicular vessels in third dimension towards the surface of the epithelium, represents premalignant and malignant lesions^8^.

In our study, the new classification introduced by ELS (2016) was used to assess vascular pattern surrounding granuloma-like lesions following TLM. Lesions characterized by perpendicular vascular pattern or intraepithelial papillary capillary loops, were identified as NBI positive, others as NBI negative.

Granulomas can be divided according to their etiology into two main subtypes: spontaneous granulomas, also known as contact granulomas, and iatrogenic granulomas^15^. In this study, we assessed only patients with iatrogenic granulomas, sequel of CO_2_ laser cordectomies. The inflammation process that is associated with infection of the perichondrium is a trigger in granuloma formation process. According to many authors, the common histological features of granulomas are identified as nonspecific reparative granulation tissue covered by hyperplastic squamous epithelium with acanthotic thickening^16,17^. In this study, the definite confirmation of the benign character of granuloma-like lesion was the granulation tissue without atypical cells in the final histology examination.

Shoffel-Havakuk *et al*.^15^ claimed that smoking is a significant risk factor in patients with iatrogenic granuloma. In our group, a significant proportion of patients 23/47(49%), who developed GLL were in fact heavy smokers. Schoffel-Havakuk *et al*.^15^ considered a pedunculated character of lesion as a common feature of iatrogenic granulomas; we confirmed this finding in our study in majority of patients (40/47).

The main feature of all granulomas is their typical location: the posterior third of the glottis. The primary laryngeal cancer in this location is very rare. If present in this site, usually is a result of cancer extension from the anterior glottic lesion^18^. Among our patients, 22/47 had granuloma in the anterior commissure, 20/47 in the posterior third of the glottis and 5/47 in middle part of the glottis. NBI positive lesions (13/47), which were confirmed in histology, were localized respectively: 8 in anterior commissure, 3 in middle part of the glottis and 2 in the posterior part of the glottis. According to these results, localization not always is a good prognostic factor to predict benign character of granuloma following TLM. Therefore, NBI can be very helpful in determination of the potential local recurrence or benign granuloma.

Iatrogenic granulomas have shorter recovery time and lower recurrence rate in comparison to spontaneous granulomas. Therefore, based on our findings, we recommend for patients after TLM with typical features for contact granulomas and without pathological vessels in NBI, conservative treatment (antireflux therapy) and close observation. According to our experience, the need for biopsy among patients after TLM should be based on clinical assessment by white light and NBI endoscopy and initial response to antireflux medication.

## Conclusions

Based on our results, NBI can reliably differentiate between postoperative laryngeal granuloma and local tumor recurrence. We recommend that in cases of typical granuloma without pathological vessels in NBI, conservative antireflux therapy should be implemented. However, these patients should remain under close and regular tumor follow-up.
