# Author Correction: Evaluation of narrow band imaging in the assessment of laryngeal granuloma

**DOI:** 10.1038/s41598-020-61339-x

**Published:** 2020-03-04

**Authors:** H. Klimza, W. Pietruszewska, J. Jackowska, K. Piersiala, M. Wierzbicka

**Affiliations:** 1 Department of Otolaryngology, Head and Neck Surgery, Poznań University of Medical Sciences, Poznań, Poland; 2 Clinical Department of Otolaryngology and Oncological Laryngology, Medical University of Lodz, Lodz, Poland; 3 Institute of Human Genetics, Polish Academy of Sciences, Strzeszynska 32, 60-479 Poznan, Poland; 4 Student Research Group at the Department of Otolaryngology, Head and Neck Surgery Poznań University of Medical Sciences, Poznań, Poland; 5 Division of ENT Diseases, Department of Clinical Sciences, Intervention and Technology, Karolinska Institutet, Stockholm Sweden

Correction to: *Scientific Reports* 10.1038/s41598-019-50699-8, published online 06 November 2019

This Article contains an error in the order of the Figures. Figures 1, 2 and 3 were published as Figures 2, 3 and 1 respectively. The correct Figures 1, 2 and 3 appear below. The Figure legends are correct.

**Figure 1:**
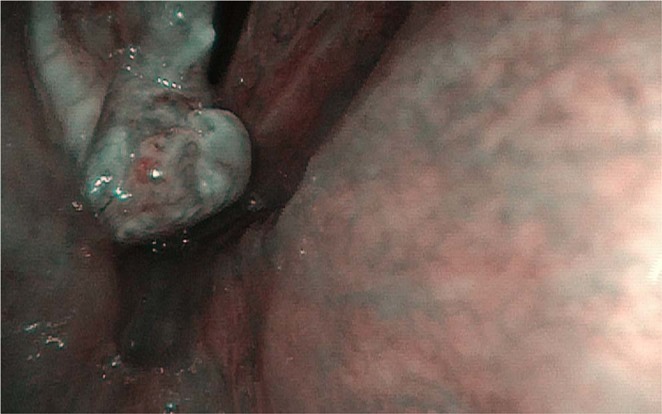
Granuloma-like lesion with the suspicious vascular pattern in NBI.

**Figure 2:**
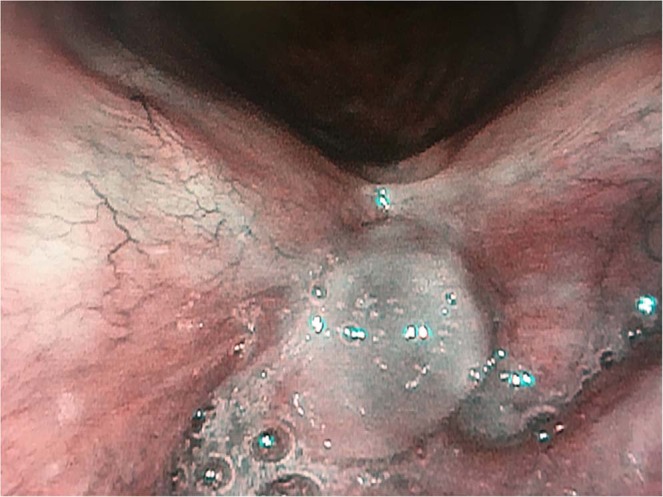
Granuloma-like lesion with the benign vascular pattern in NBI.

**Figure 3:**
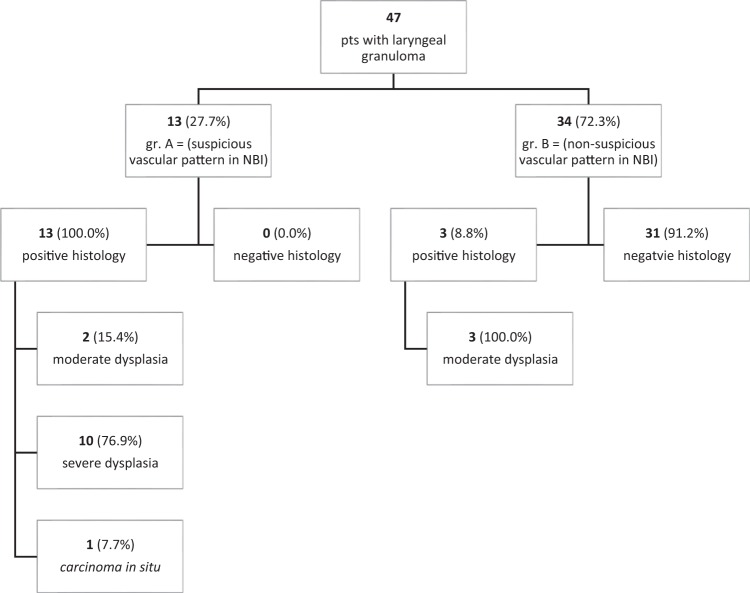
The summary of results.

